# Increased BMSC exosomal *miR-140-3p* alleviates bone degradation and promotes bone restoration by targeting *Plxnb1* in diabetic rats

**DOI:** 10.1186/s12951-022-01267-2

**Published:** 2022-03-02

**Authors:** Ning Wang, Xuanchen Liu, Zhen Tang, Xinghui Wei, Hui Dong, Yichao Liu, Hao Wu, Zhigang Wu, Xiaokang Li, Xue Ma, Zheng Guo

**Affiliations:** 1 Department of Orthopaedics, Tangdu Hospital, Fourth Military Medical University, Xi’an, 710038 Shaanxi China; 2 Department of Nutrition, Tangdu Hospital, Fourth Military Medical University, Xi’an, 710038 Shaanxi China; 3 Department of Orthopedics, The 63750 Hospital of People’s Liberation Army, Xi’an, 710043 Shaanxi China; 4 Department of Pharmacology, School of Pharmacy, Fourth Military Medical University, Xi’an, 710032 Shaanxi China

**Keywords:** Bone defect, Diabetes mellitus, Exosomes, *MiR-140-3p*, Plexin B1

## Abstract

**Background:**

Diabetes mellitus (DM) is considered to be an important factor for bone degeneration disorders such as bone defect nonunion, which is characterized by physical disability and tremendous economy cost to families and society. Exosomal *miRNAs* of BMSCs have been reported to participate in osteoblastogenesis and modulating bone formation. However, their impacts on the development of bone degeneration in DM are not yet known. The role of *miRNAs* in BMSCs exosomes on regulating hyperglycemia bone degeneration was investigated in the present study.

**Results:**

The osteogenic potential in bone defect repair of exosomes derived from diabetes mellitus BMSCs derived exosomes (DM-Exos) were revealed to be lower than that in normal BMSCs derived exosomes (N-Exos) in vitro and in vivo. Here, we demonstrate that *miR-140-3p* level was significantly altered in exosomes derived from BMSCs, ADSCs and serum from DM rats. In in vitro experiments, upregulated *miR-140-3p* exosomes promoted DM BMSCs differentiation into osteoblasts. The effects were exerted by *miR-140-3p* targeting *plxnb1*, plexin B1 is the receptor of semaphoring 4D(Sema4D) that inhibited osteocytes differentiation, thereby promoting bone formation. In DM rats with bone defect, *miR-140-3p* upregulated exosomes were transplanted into injured bone and accelerated bone regeneration. Besides, *miR-140-3p* in the exosomes was transferred into BMSCs and osteoblasts and promoted bone regeneration by targeting the plexin B1/RohA/ROCK signaling pathway.

**Conclusions:**

Normal-Exos and miR-140-3p overexpressed-Exos accelerated diabetic wound healing by promoting the osteoblastogenesis function of BMSCs through inhibition plexin B1 expression which is the receptor of Sema4D and the plexin B1/RhoA/ROCK pathway compared with diabetes mellitus-Exos. This offers a new insight and a new therapy for treating diabetic bone unhealing.

**Graphical Abstract:**

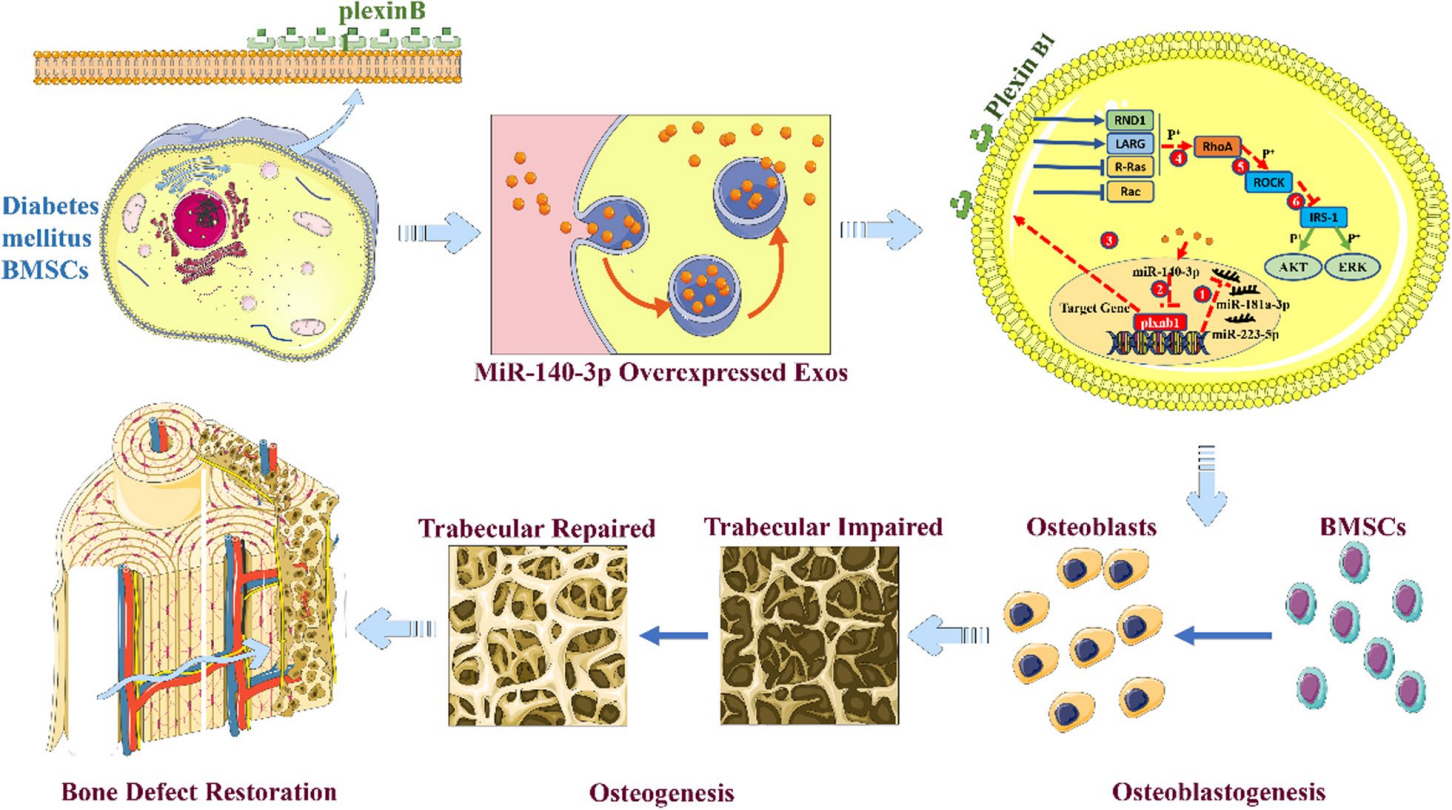

**Supplementary Information:**

The online version contains supplementary material available at 10.1186/s12951-022-01267-2.

## Introduction

Type 2 diabetes mellitus (T2DM) is a common metabolic disorder characterized by chronic hyperglycemia. According to the World Health Organization (WHO), more than 463 million people around the world are currently suffering from diabetes mellitus (DM), of which T2DM accounts for 90% [1]. There is accumulating evidence of a strong interaction between glucose levels and changes in bone metabolism. Impaired bone healing is considered an important complication associated with DM. T2DM also results in changes in bone metabolism detrimental to bone quality and a cause of reduced bone strength, increased fracture risk, and impaired bone healing [2–4]. It has been reported that the duration of fracture healing in DM patients is prolonged by 87% [5]. Bone regeneration is often delayed in DM patients, most likely due to impaired osteogenic differentiation [6, 7]. Furthermore, the inhibition of transcription factor expression, which is crucial for the development of an osteoblastic phenotype such as runt-related transcription factor 2 (Runx-2), and decreased cell proliferation and growth factor expression contribute to impaired bone healing [8]. Despite such evidence, the precise mechanisms causing bone impairment in T2DM are not yet fully understood. Further investigation is required to elucidate the pathophysiology of diabetic bone and develop successful strategies to treat this growing medical complication that has led to considerable socioeconomic burden.

Bone mesenchymal stem cells (BMSCs), also known as bone marrow-derived mesenchymal stem cells (MSCs), are considered a promising source of cells for tissue engineering because they can stimulate osteogenesis required for bone regeneration [9, 10]. Recent evidence indicates that the osteogenic capability of BMSCs is reduced in DM patients in comparison with that in normal populations [11]. The ability of BMSCs to self-renew and their therapeutic potential play important roles in regulating the healing of bone defects [10]. Recently, multiple studies have revealed that the efficacy of MSC-based therapies for bone defect regeneration is attributable to their exosome-mediated autocrine and paracrine actions [12–15]. MSC-derived exosomes are more stable for therapeutic interventions in particular physiological circumstances due to the additional immune privilege compared with stem cells [16, 17]. Extensive studies have demonstrated that BMSC-derived exosomes can stimulate the proliferation and osteogenic differentiation of BMSCs, thereby promoting osteogenesis, angiogenesis, and bone mineralization within bone defects [18, 19], suggesting that they could have potential as a cell-free therapy for bone defect healing.

Exosomes are extracellular vesicles, ranging in diameter from 30 to 150 nm and thought to be the primary mediators of intercellular communication, by means of small RNA molecules, especially microRNAs (*miRNAs*) [20–23]. A number of bone-derived exosomal *miRNAs* that regulate bone remodeling have been characterized. Exosomes are found in the circulation and contain numerous miRNA molecules which perform important regulatory roles at both local and distal sites. It has been revealed that BMSC-derived exosomes promote osteoblastogenesis via *miR-196a *in vitro and have been shown to increase bone regeneration in calvarial defects in a Sprague–Dawley rat model [18]. A previous study found that osteoclast-derived exosomal *miR-214-3p* can be transferred to osteoblasts, inhibiting osteogenic activity and bone formation. However, inhibition of *miR-214-3p* expression in osteoclasts promoted osteogenesis and bone regeneration [24]. Moreover, *miR-31a-5p* expression increased significantly in BMSCs and BMSCs-derived exosomes from elderly rats. However, BMSCs in which *miR-31a-5p* was overexpressed displayed increased adipogenesis and decreased osteogenesis. Inhibition of *miR-31a-5p* prevented substantial bone loss and decreased osteoclastic activity in elderly rats [25]. In addition, *miR-100-5p* derived from MSCs harvested from the infrapatellar fat pad (IPFP) was found to reduce autophagy in chondrocytes to a significant extent by inhibition of the mTOR autophagy pathway, protecting OA mice and normalizing cartilage homeostasis [26].

The results described above suggest that DM may inhibit BMSC differentiation by modification of *miRNA* expression, which modulates osteogenesis. In the present study, we found that exosomes secreted by BMSCs derived from type 2 diabetic rats (DM-Exos) delayed the healing of bone defects in normal rats while those from normal rats (N-Exos) promoted bone formation in diabetic rats. In addition, DM-Exos reduced levels of mineral deposits while N-Exos increased mineralization by BMSCs in vitro. The expression of *miR-140-3p* in exosomes derived from type 2 diabetic rat BMSCs was lower than that in normal rat BMSCs when analyzed by high-throughput sequencing. Furthermore, *plxnb1* was found using TargetScan software to be the downstream target of exosomal *miR-140-3p*. Notably, *miR-140-3p* overexpression or inhibition modulated *plxnb1* expression and significantly enhanced BMSC differentiation or impeded BMSC osteoblastogenesis. Thus, a mixture of matrigel and exosomes overexpressing *miR-140-3p* significantly enhanced bone formation compared with N-Exos and DM-Exos treatment groups. Taken together, the present study revealed a role for BMSC-derived exosomal *miR140-3p* in the regulation of DM-associated impaired bone healing, suggesting that *plxnb1* may be a promising target for miR-140-3p.

## Results

### BMSCs secrete exosomal miRNAs

**Fig. 1:**
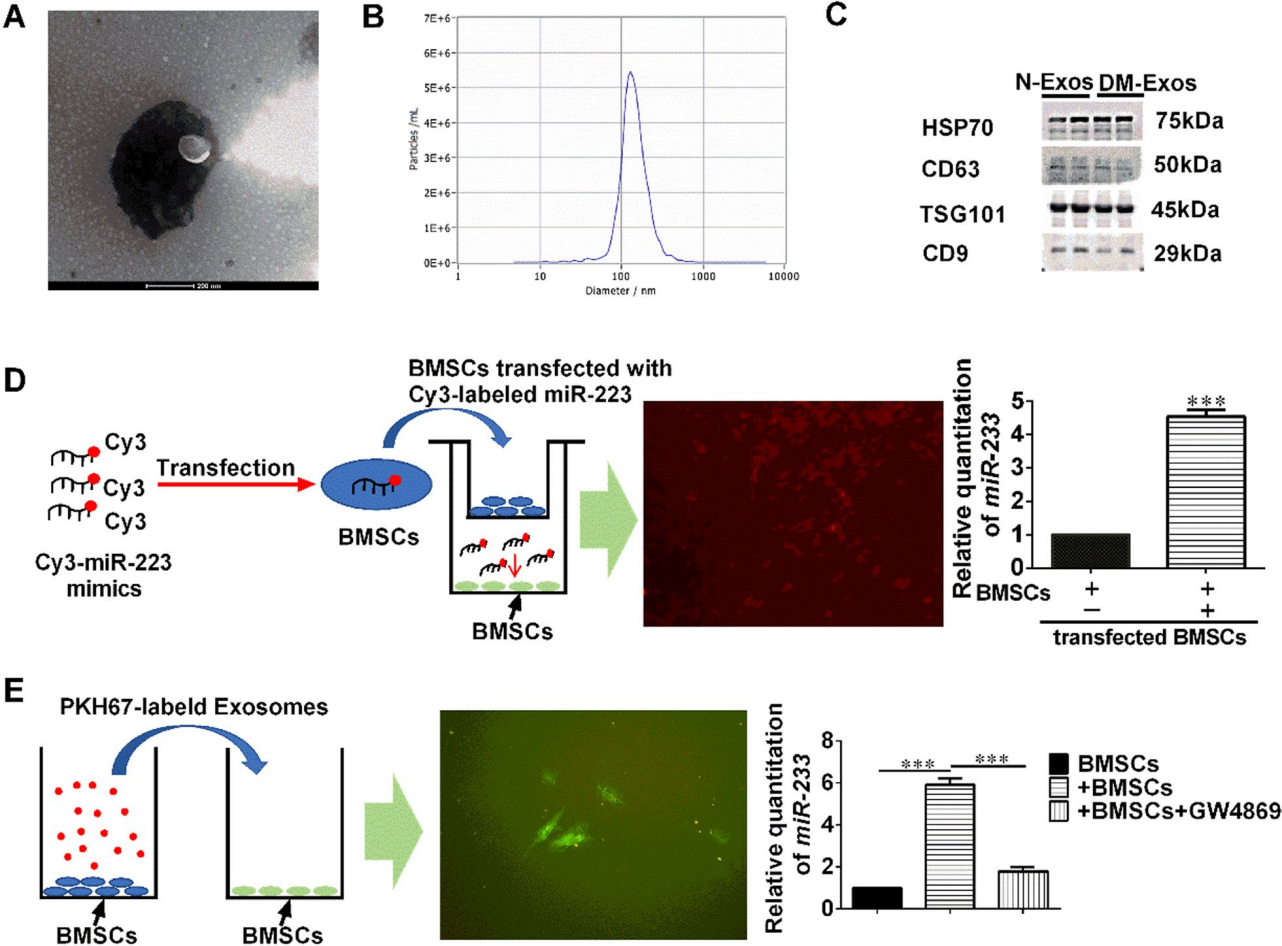
BMSCs Secrete Exosomal miRNAs. **A** Electron microscopic analysis of Exos secreted by BMSCs. Scale bar: 200 nm. **B** Particle size distribution of vesicles secreted from BMSCs, as measured by NanoSight analysis. **C** The concentrations of the exosome (Exo)-specific marker TSG101 and extracellular vesicle-related protein markers HSP70, CD63, and CD9 were measured by Western blot analysis. The blots are representative of 3 replicates of independent experiments, each with 2 samples. **D** BMSCs transfected with a Cy3-labeled miR-223 mimic were co-cultured with BMSCs in a transwell (membrane pore size: 0.4 μm) plate. **E** Exos from BMSCs were labeled with PKH26 and then added to BMSC cultures. The results demonstrate the effects of the EV secretion inhibitor GW4689 (10 mM) on exosome-dependent miRNA delivery from BMSCs into recipient BMSCs. Data represent means ± SD. ***p < 0.001

Firstly, BMSC-derived exosomes were isolated, their contents identified, and their morphology assessed by the expression of specific markers and by TEM, respectively. The exosomes were isolated from BMSCs (CD29^+^Sca-1^+^) from the bone marrow cells of 3-month-old normal rats (N-rats) and diabetic rats (DM-rats). The two groups of BMSCs were then cultured in exosome-free medium for 72 h, from which conditioned medium was collected, then dead cells and debris were removed. Ultracentrifugation was used to isolate the BMSC-derived extracellular particles. The morphology of the isolated extracellular particles was observed by TEM, revealing that they were round, lipid-bilayered vesicles (Fig. 1A), confirming that they were exosomes. A nanoparticle tracking system was used to measure the concentration and size distribution of the purified particles. There were 5.4 × 10^6^ with a mean diameter of 133 nm (Fig. 1B). The expression of the exosome-specific protein markers HSP70, TSG101, CD63, and CD9 identified by Western blotting analysis also demonstrated that the extracellular particles isolated from N-rats and DM-rats were exosomes (Fig. 1C). In addition, there were no differences between N-rats and DM-rats in the expression of the exosome-associated protein markers. The results above suggest that the BMSC-derived extracellular particles were exosomes.

Whether BMSCs could secrete extracellular miRNAs able to be phagocytized by target cells was subsequently determined. *MiR-223* is specific for myeloid cells and so was used as a marker. BMSCs were transfected with fluorescent Cy3-labeled *miR-223* mimics then co-cultured with untransfected BMSCs seeded in the upper and lower wells of a transwell plate, respectively, for 12 h (Fig. 1D). Red fluorescent Cy3 dye in the untransfected BMSCs demonstrated that the Cy3-*miR-223* mimic was transferred from the BMSCs in the upper well to those in the lower well (Fig. 1D). The quantities of *miR-223* in the BMSCs in the lower well increased by almost fivefold following co-culture. In addition, BMSCs were also cultured with Cy3 alone (without miR-223), and little red fluorescence was observed in these cells (Cy3-BMSCs), while no fluorescence was observed in BMSCs seeded in the lower well when co-cultured with the Cy3-BMSCs (Additional file 1: Fig. S1). Taken together, the results indicate that BMSCs can secrete extracellular miRNAs that can be absorbed by other cells such as BMSCs.

Finally, whether BMSC-derived exosomes could be absorbed by other BMSCs was determined. BMSCs-derived exosomes were labeled with the fluorescent dye PKH67 and then added to a fresh culture of BMSCs. After 12 h, the BMSCs fluoresced green, indicating that the exosomes were absorbed by the BMSCs (Fig. 1E). In addition, a transwell experiment was also performed to verify whether BMSCs can secret miRNA-containing exosomes to other cells. BMSCs seeded in the lower well expressed an approximately sixfold increase in *miR-223* when co-cultured with BMSCs seeded in the upper well. However, the extracellular vesicle secretion inhibitor GW4869 reversed the increase in *miR-223* expression in BMSCs seeded in the lower well when co-cultured with BMSCs seeded in the upper well (Fig. 1E). All the experiments demonstrated that BMSCs secrete *miRNAs* containing exosomes that could be delivered to target cells.

### Differential effects of exosomes derived from DM and normal rats on the restoration of bone defects

BMSCs play a vital role in bone remodeling. There is considerable evidence indicating that the ability of BMSCs to differentiate into osteoblasts declines in patients with diabetes compared with those without. Therefore, the ability of exosomes secreted by DM-BMSCs and N-BMSCs to modulate bone formation in vivo was tested.

**Fig. 2:**
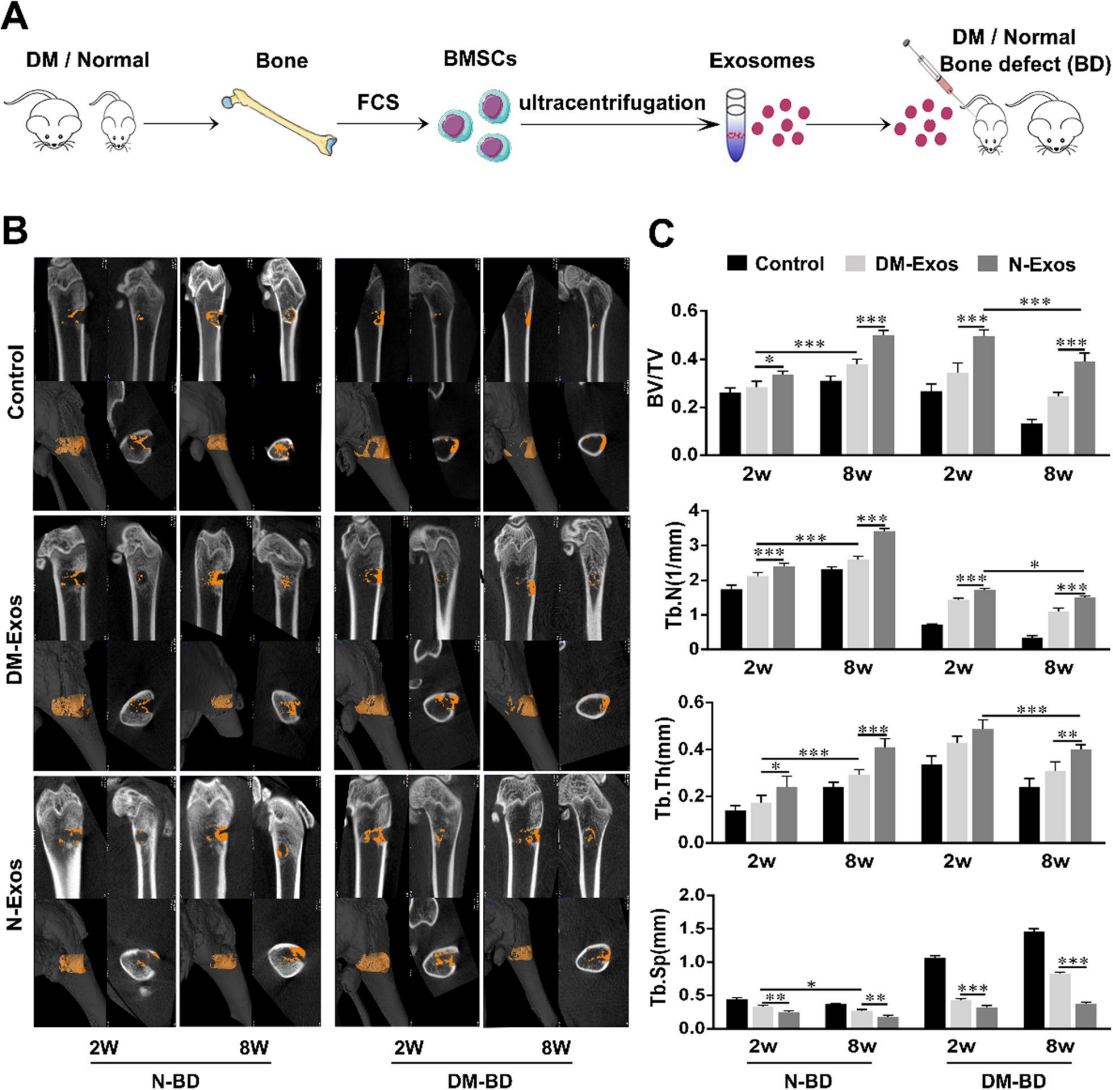
Impaired bone formation in rat bone defect caused by DM-Exos. **A** N-Exos or DM-Exos were implanted into bone defects of rats that were normal or suffered diabetes mellitus. **B** Representative micro-CT images within a region of interest (ROI) representing bone defects 2 and 8 weeks after surgery in normal rats and those with diabetes mellitus. n = 5 in each group. **C** Quantitative analysis of micro-CT imgaes of the new bone formation in bone defects 2 and 8 weeks after transplantation with N-Exos and DM-Exos. BV: bone volume; TV: tissue volume; Tb,N: trabecular number; Tb.Th: trabecular thickness; Tb.Sp: trabecular Spacing. n = 5 in each group; *p < 0.05; **p < 0.01; ***p < 0.001. Data represent means ± SD

Both normal and diabetic rats with bone defects received N-Exos (240 μg) or DM-Exos (240 μg) derived from normal rat BMSCs or DM rat BMSCs (Fig. 2A). Micro-CT was used to quantify the bone mass within the defect in each group (Fig. 2B, C). The results demonstrate that although treatment with exosomes increased bone mass, N-Exos caused the formation of a greater quantity of bone than DM-Exos after 2 and 8 weeks, as shown by the difference in BV/TV values. In defects receiving DM-Exos, the BV/TV in 8 weeks was higher than that in 2 weeks. Similarly, trabecular number and trabecular thickness also increased due to the transplantation of exosomes compared with the control group, while treatment with N-Exos increased trabecular number and thickness compared with rats treated with DM-Exos. Furthermore, the trabecular number and trabecular thickness after 8 weeks were also higher than after 2 weeks in the DM-Exos group. The trabecular spacing decreased in the exosome-treated groups compared with the control group. The data above indicate that although exosomes promoted bone formation, DM-Exos impaired bone formation compared with the N-Exos treatment in both normal rats and those with diabetes mellitus.

### Effect of N-Exos and DM-Exos on bone regeneration in a rat bone defect model

**Fig. 3:**
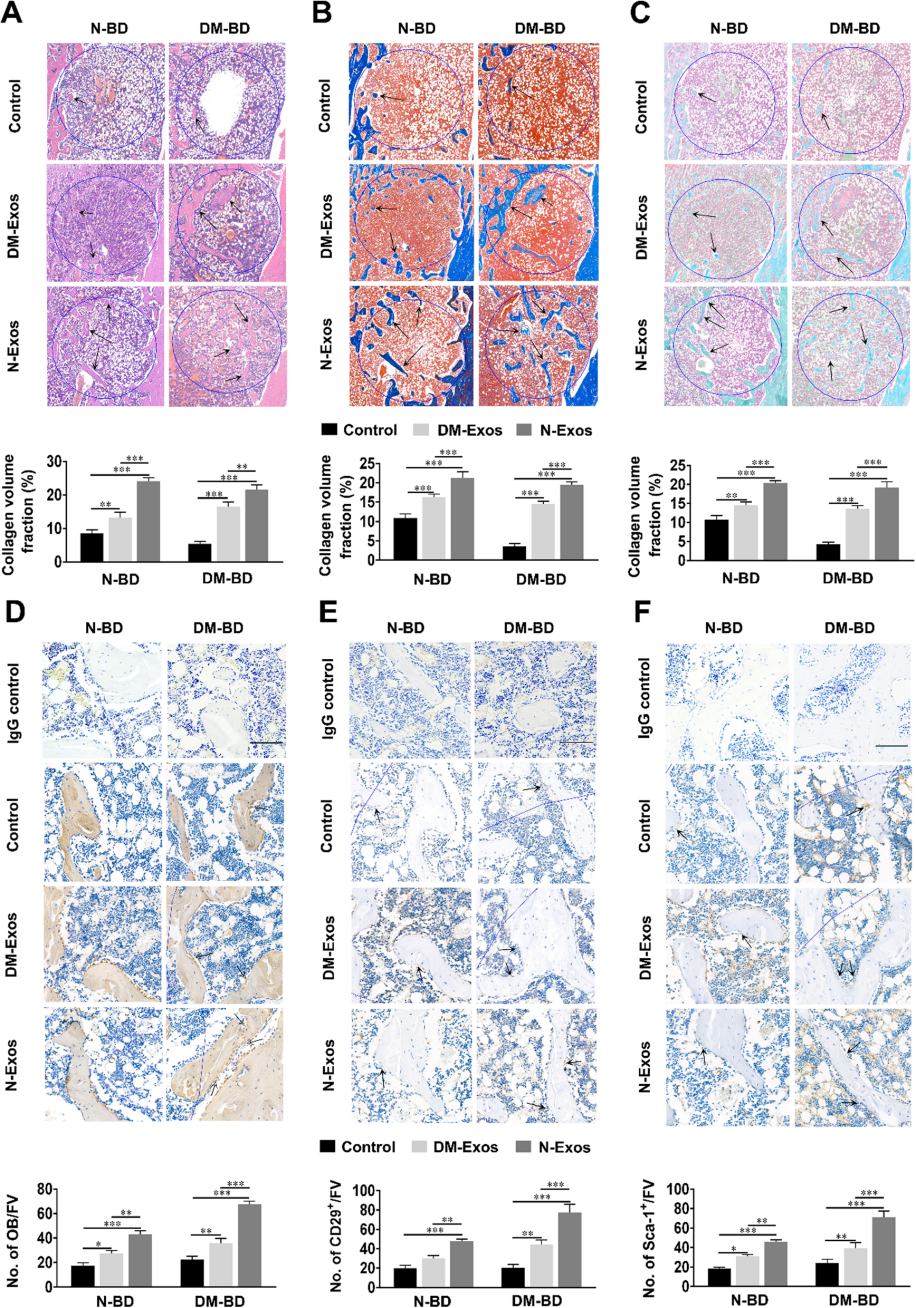
Histological/Immunohistological analysis of bone formation after transplantation of exosomes. **A**–**C** Histological analysis of decalcified sections in both normal and diabetic rats. HE/Masson’s/ Safranin O-fast green staining of new bone formation in the bone defect. The black arrow indicates new bone in light pink, blue, or green within the circle, indicating the 3 mm-diameter defect. New bone formation in the bone defects was analyzed using ImageJ software. n = 5 for each group. **D**–**F** Immunohistological analysis of the osteoblasts and BMSCs in both normal and diabetic rats. Collagen-1 and CD29/Sca-1 staining of osteoblasts and BMSCs in bone defects. Immunohistochemical staining with IgG was as the control group. Black arrows indicate osteoblasts and BMSCs on the bone surface. n = 5 in each group; *p < 0.05; **p < 0.01; ***p < 0.001. Data represent means ± SD

Histological analysis and immunohistochemical staining were utilized to demonstrate the different effects of exosomes isolated from DM and normal rats on bone regeneration and mineralization. Collagen is a constituent of mineralized bone. In hematoxylin and eosin (HE)-stained sections (Fig. 3A), collagen can be visualized as light pink tissue. In the bone defect of rats, little mineralized bone tissue was observed in the DM-Exos transplanted group in comparison with the N-Exos treatment group, although more than in the control group. Collagen in bone is visualized as blue tissue in Masson’s trichrome-stained sections (Fig. 3B). In normal rats with bone defects, the volume of mineralized bone tissue in the DM-Exos transplanted group was less than in the N-Exos group. The volume of mineralized bone tissue in untreated bone defect in normal rats was less than that in the exosome-treated group. Consistent with the results of bone defects in DM rats, the volume of mineralized bone tissue in the N-Exos transplanted group was greater than that observed in the DM-Exos group. In addition, the volume of mineralized bone tissue in the control group was less than in the Exos-treated groups. In Safranin O/fast-green stained sections (Fig. 3C), the collagen was identified as being green. The volume of mineralized bone stained green was lower in the DM-Exos group than in the N-Exos group. Moreover, the mineralized bone volume of the control group was the smallest of the comparative groups. Immunohistochemical staining with IgG was regarded as negative control. Figure 3D showed that rats with bone defects had fewer osteoblasts in the control group than in either the DM-Exos or N-Exos group, while the numbers in the N-Exos group were higher than in the DM-Exos group. BMSCs can differentiate into osteoblasts and the CD29/Sca-1 are the specific markers for BMSCs. In the bone defects of normal rats (Fig. 3E, F), the number of BMSCs on the bone surface in the DM-Exos treatment group was less than in the N-Exos group, which was more than in the control group. The numbers of BMSCs on the bone surface in the N-Exos treatment group in DM rats was considerably greater than in the DM-Exos treatment group, which was more than in the control group. The data above indicate that N-Exos promoted bone formation via increasing the numbers of BMSCs and osteoblasts in DM rats, resulting in bone healing.

To further clarify the effect of exosomes on BMSC osteoblastogenesis in vitro, calcium deposition by N-BMSCs/DM-BMSCs treated with DM-Exos or N-Exos was investigated (Additional file 2: Fig. S2 A). DM-Exos and N-Exos were derived from the BMSCs of DM and normal rats. The exosomes were cultured with BMSCs for 14 days, in each case. Calcium deposition was assessed using Alizarin red S staining. The results demonstrate that mineralization was significantly enhanced following treatment with both DM-Exos and N-Exos, although N-Exos treatment exhibited greater osteogenesis than DM-Exos treatment in both N-BMSC and DM-BMSC groups (Additional file 2: Fig. S2 B,C). The results suggest that DM-Exos decreased osteoblastogenesis and calcium deposition in N-BMSCs while N-Exos increased osteoblastogenesis and calcium deposition in DM-BMSCs. N-Exos displayed a greater promotion of osteogenesis in DM-BMSCs than the treatment of N-BMSCs with DM-Exos.

### Exosomal miRNAs promote the osteogenesis of BMSCs

**Fig. 4:**
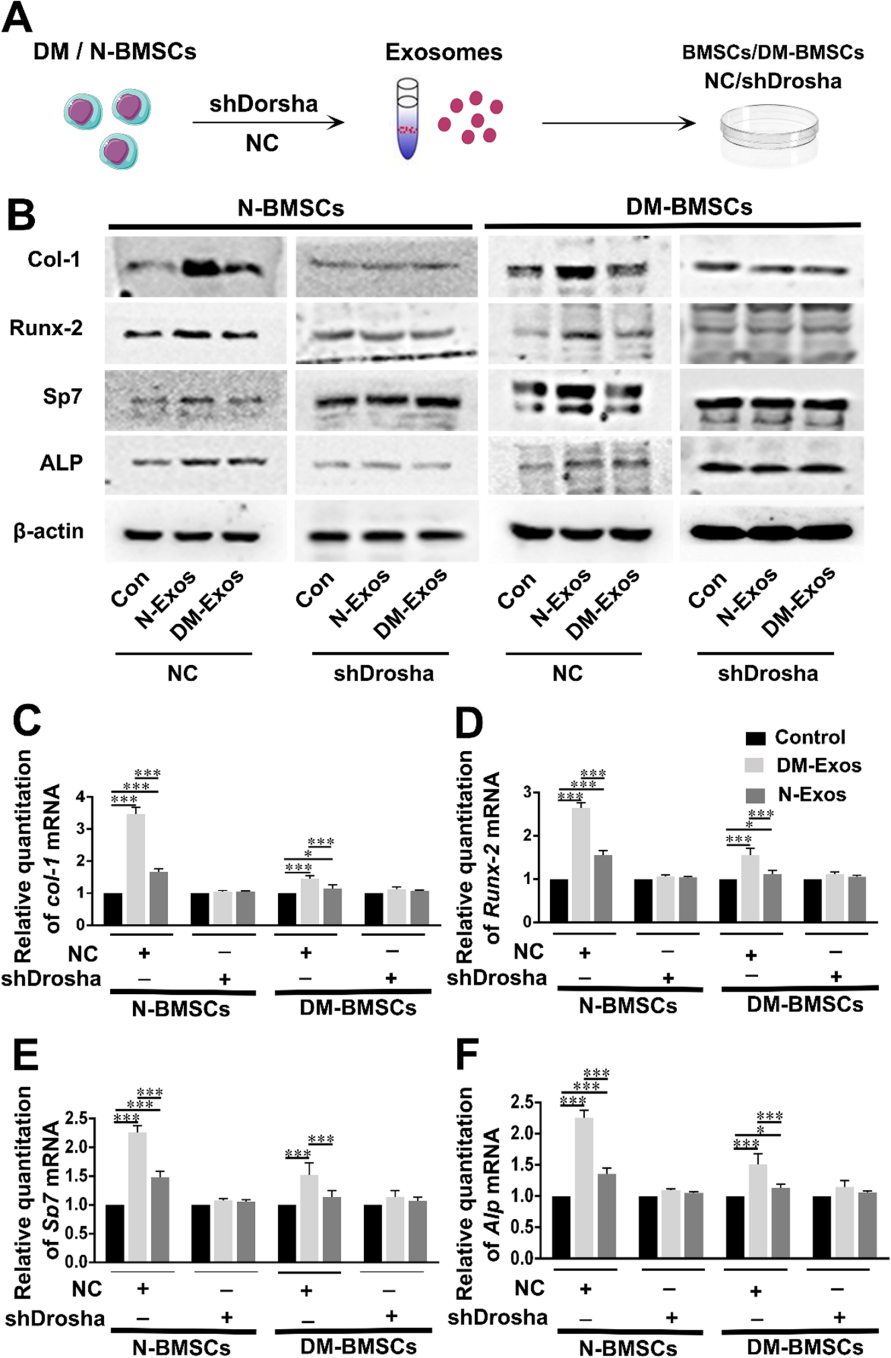
Exosomes modulate BMSC differentiation through exosomal miRNAs. **A** Exos were collected from normal BMSCs and DM BMSCs after siRNA-mediated knockdown of Drosha and then cultured with untreated N-BMSCs or DM-BMSCs. After 48 h, the N-BMSCs and DM-BMSCs were collected and the protein expression levels of osteogenic molecules were measured by Western blotting. **B** Western blot analysis of Col-1, Runx-2, ALP, and Sp7 expression in N-BMSCs and DM-BMSCs cultured with Exos both with or without Drosha knockdown. n = 3 in each group. **C**–**F** Quantitative analysis of Col-1, Runx-2, ALP, and Sp7 expression by Western blotting. n = 3 in each group. *p < 0.05; **p < 0.01; ***p < 0.001. Data represent means ± SD

Given the negative impact of DM-Exos on bone formation and the positive effect of N-Exos on osteogenesis, the role of exosomal miRNA molecules in the osteogenesis of BMSCs was next evaluated. As is well known, exosomes are a type of extracellular vesicle containing miRNA molecules secreted from various cells. Circulating exosomes are considered an important mechanism for the transport of miRNA. To demonstrate that miRNAs are key functional components for osteogenesis to occur, a small interfering RNA (siRNA) molecule was used to knock down Drosha to inhibit miRNA synthesis and so deplete the secreted exosomes of miRNA molecules (Fig. 4A). Firstly, N-BMSCs were treated with exosomes isolated from DM-BMSCs and N-BMSCs. Consistent with the previous results, the exosomes promoted Col-1, Runx-2, Sp7, and ALP expression, with greater osteogenesis caused by N-Exos than DM-Exos. In contrast, the treatment of N-BMSCs with exosomes isolated from DM-BMSCs and N-BMSCs in which Drosha had been knocked down demonstrates that these exosomes did not significantly promote osteogenesis to a greater extent than the control group. These results indicate that miRNAs are responsible for the promotion of osteogenesis by exosomes. Consistent with the results above, exosomes without knockdown promoted Col-1, Runx-2, Sp7, and ALP expression in DM-BMSCs, although the N-Exos treatment group exhibited greater osteogenesis than the DM-Exos group. Conversely, the treatment of DM-BMSCs with exosomes isolated from N-BMSCs and DM-BMSCs that had undergone Drosha-knockdown indicated that the ability of the exosomes to promote osteogenesis in DM-BMSCs had disappeared (Fig. 4B–F). These results indicate that miRNA molecules perform a critical role in the osteogenesis of exosomes.

Taken together, the results suggest that N-BMSCs secrete exosomal miRNAs in their normal state that provide protection against diabetes-induced bone loss.

### Hyperglycemia induces changes in BMSC-Exo miRNA expression

**Fig. 5:**
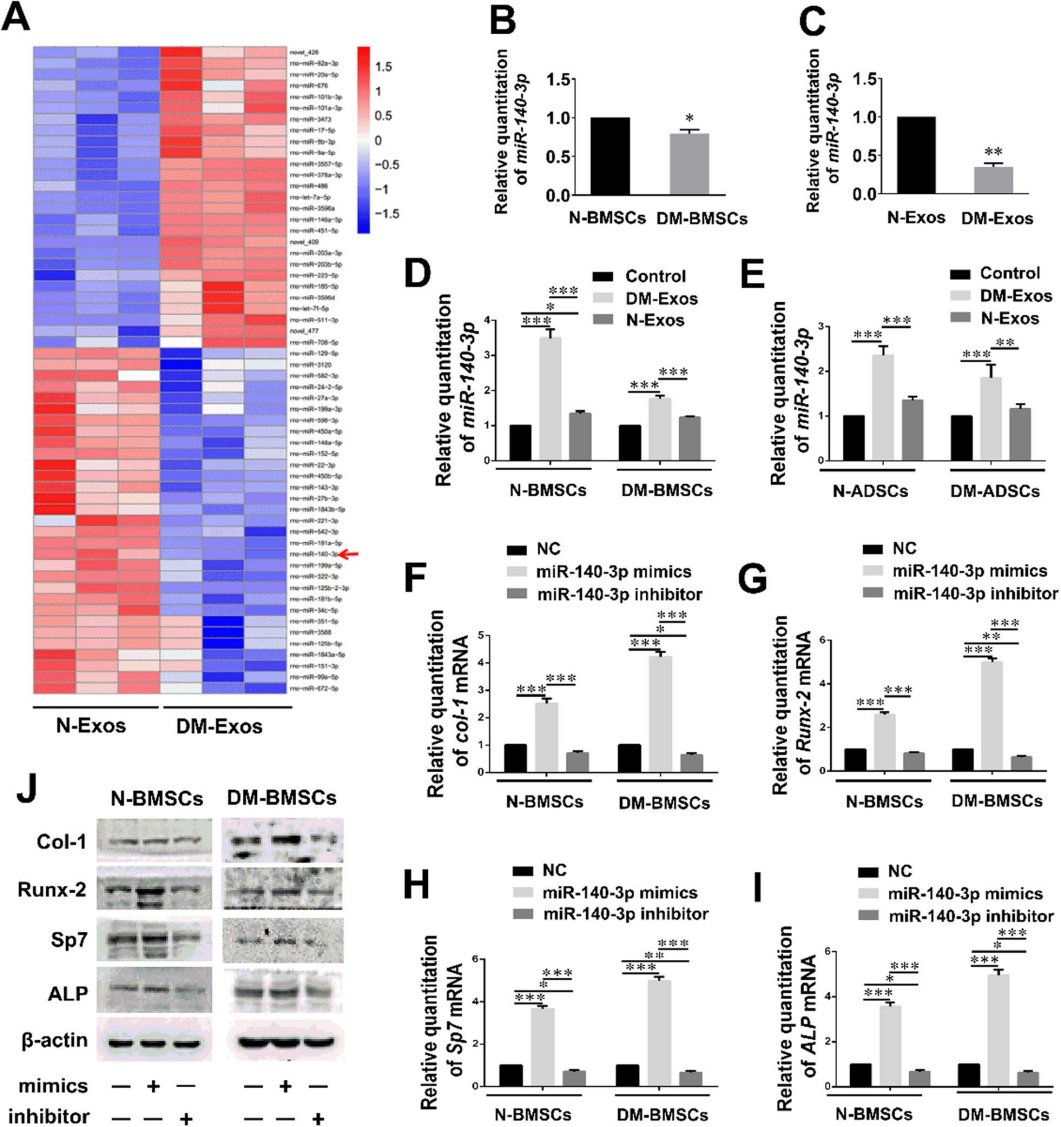
Diabetes results in changes in the expression of miRNA in BMSCs-Exos. **A** Differential expression levels of exosomal miRNAs between N-Exos and DM-Exos. MiR-140-3p is an exosomal miRNA that underwent significantly reduced expression in DM-Exos compared with N-Exos. **B** Differential expression levels of miR-140-3p between N-BMSCs and DM-BMSCs. n = 3 in each group. **C** Expression levels of miR-140-3p between N-Exos and DM-Exos. n = 3 in each group. **D**, **E** MiR-140-3p expression in N-BMSCs/ADSCs and DM-BMSCs/ADSCs, which were treated with N-Exos and DM-Exos. n = 3 in each group. **F**–**I** qPCR examination of mRNAs levels of *Col-1, Runx-2, ALP*, and *Sp7* in N-BMSCs and DM-BMSCs after miR-140-3p NC/mimics/inhibitor treatment. n = 3 in each group. **J** Western blot analysis of Col-1, Runx-2, ALP and Sp7 expression in N-BMSCs and DM-BMSCs after miR-140-3p NC/mimics/inhibitor treatment. n = 3 in each group. *p < 0.05; **p < 0.01; ***p < 0.001. Data represent means ± SD

Given the negative osteogenic effects of exosomes from DM-BMSCs compared with those from N-BMSCs, DM-induced changes in the expression of miRNA molecules in BMSCs were then assessed. Deep sequencing of small RNAs from normal and DM BMSC-Exos was conducted to identify the differential expression of miRNAs. More than 600 miRNAs were identified in the exosomes of DM-BMSCs and N-BMSCs. Considerable differences in the miRNA expression of DM-Exos and N-Exos were observed. Of the differentially-expressed miRNAs, those most significantly differentially expressed in DM-Exos compared with N-Exos are presented in Fig. 5A.

Among the miRNAs identified here, *miR-140-3p*, *miR-34c-5p*, *miR-99a-5p*, and *miR-27a-3p* were expressed in DM-Exos to a significantly lower extent than in N-Exos, and so were selected to evaluate the ability of miRNAs to influence osteogenesis in DM-BMSCs (Additional file 3: Fig. S3). Mimics and inhibitors of the five *miRNAs* were evaluated to investigate their effect on osteoblastogenesis in DM-BMSCs. The results demonstrate that *miR-140-3p* significantly promoted osteogenesis in DM-BMSCs in contrast to the other *miRNAs*. Additionally, *miR-140-3p* has been previously reported to have anti-inflammatory effects [27] and so the results suggest that *miR-140-3p* probably plays an important role in modulating the differentiation of BMSCs in DM.

*MiR-140-3p* expression in N-BMSCs was greater than in DM-BMSCs (Fig. 5B), resulting in increased levels of *miR-140-3p* being present in N-Exos (Fig. 5C). Incubation of N-BMSCs, DM-BMSCs, normal adipocyte-derived stem cells (N-ADSCs), and DM-ADSCs with N-Exos resulted in a significant increase in the expression of *miR-140-3p* within these cells, while treatment with DM-Exos led to a small or insignificant effect (Fig. 5D, E). In addition, N-BMSCs were treated with *miR-140-3p* mimics/inhibitor to determine their effects on osteogenesis. qPCR analysis demonstrated the *Col-1, Runx-2, Sp7*, and *ALP* expression in the *miR-140-3p* mimics treatment group being significantly higher than in the control and inhibitor groups. Furthermore, there was no significant difference between the control and inhibitor groups (Fig. 5F–I). Western blotting analysis yielded results consistent with the results described above (Fig. 5J). *MiR-140-3p* mimics significantly promoted Col-1, Runx-2, Sp7, and ALP protein expression levels compared with those in the control and inhibitor groups. Additionally, no significant difference between the control and inhibitor groups was observed.

### *MiR-140-3p promotes osteoblastogenesis in BMSCs *via* inhibition of the plexin B1/RhoA signaling pathway*

**Fig. 6:**
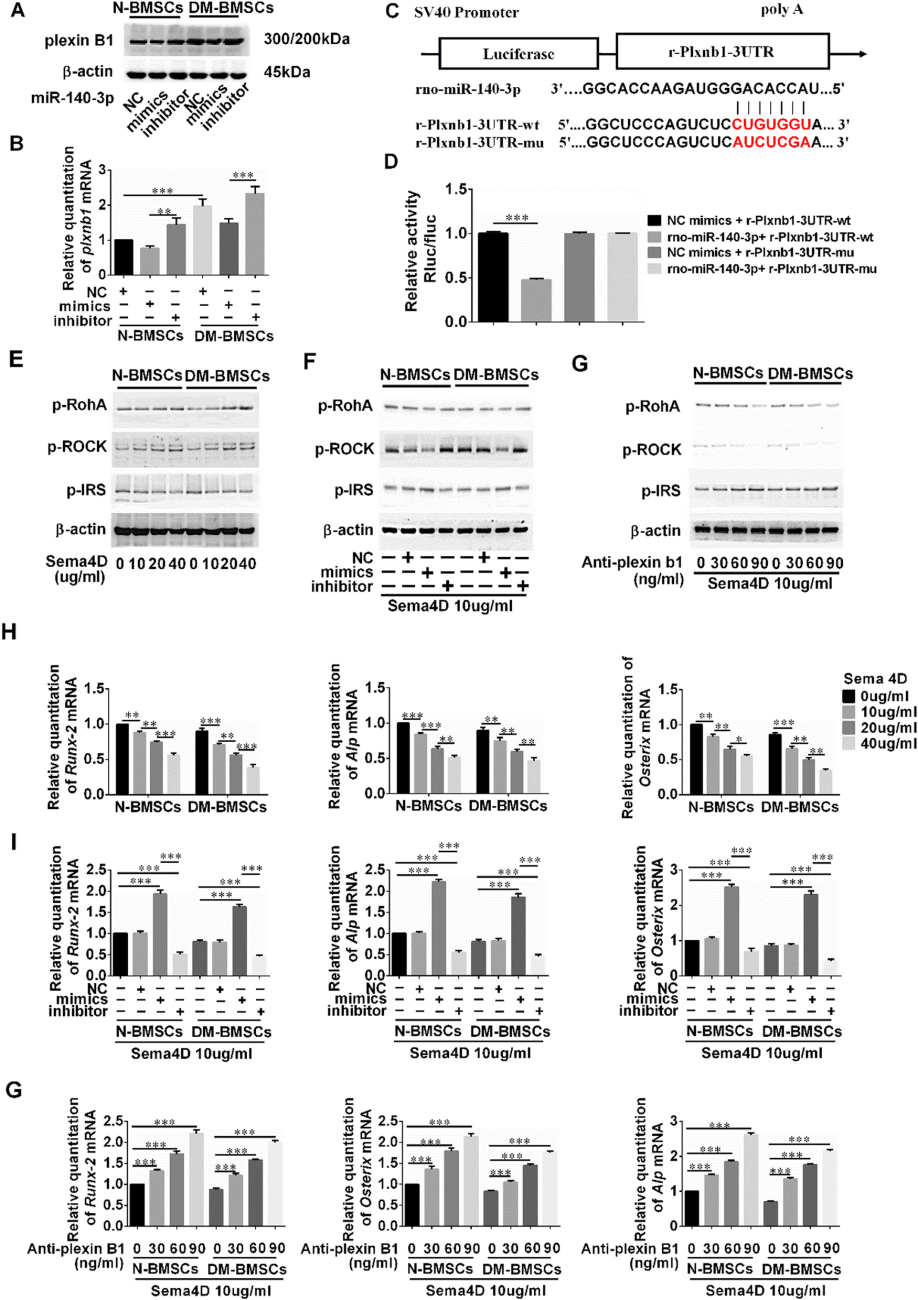
*MiR-140-3p* regulation of BMSC differentiation via Plexin B1. **A**, **B** Plexin B1 plays an important role in the interaction between osteoblasts and osteoclasts. The expression of plexin B1 in N-BMSCs and DM-BMSCs was examined using Western blot analysis and qPCR after treatment with *miR-140-3p* NC/mimics/inhibitor. n = 3 in each group. **C** Schematic illustration of the sequences for *miR-140-3p* and the WT or mutated 3′-UTR of *plxnb1* mRNA. **D** Dual Rluc/Fluc luciferase luminescence intensity of *plxnb1* WT or mutated 3′-UTR reporter plasmids in HEK293 cells co-transfected with *miR-140-3p* mimics or miR-NC mimics. **E** Protein expression levels of p-RhoA, p-ROCK, and p-IRS in N-BMSCs and DM-BMSCs after Sema4D treatment (0, 10, 20, 40 μg/mL). **F** Levels of p-RhoA, p-ROCK, and p-IRS in N-BMSCs and DM-BMSCs transfected with *miR-140-3p* NC/mimics/inhibitor after treatment with Sema4D (10 μg/mL) as determined by Western blotting. **G** Levels of p-RhoA, p-ROCK, and p-IRS in N-BMSCs and DM-BMSCs as measured by Western blotting, after culture with anti-plexin B1 (0, 30, 60, 90 ng/mL) and Sema4D (10 μg/mL). **H** Levels of *Runx-2, ALP*, and *osterix* in N-BMSCs and DM-BMSCs after treatment with Sema4D (0, 10, 20, 40 μg/mL). **I** qPCR analysis of *Runx-2, ALP,* and *osterix* expression levels in N-BMSCs and DM-BMSCs transfected with miR-140-3p NC/mimics/inhibitor and cultured with Sema4D (10 μg/mL). **J** mRNAs expression levels of *Runx-2, ALP,* and *osterix* in N-BMSCs and DM-BMSCs treated with anti-plexin B1 (0, 30, 60, 90 ng/mL) and Sema4D (10 μg/mL). Data represent means ± SD (n = 3 in each group. Western blotting and qPCR experiments were repeated 3 times). *p < 0.05; **p < 0.01; ***p < 0.001

To further explore the mechanism by which N-Exos induces osteogenesis, the influence of *miR-140-3p* on osteoblast differentiation signaling was determined. *MiRNAs* inhibit target gene functions by binding to the 3′ untranslated region (3′-UTR) or protein-coding sequence of specific mRNAs, assisting to degrade the mRNA or suppress translation. Bioinformatic software TargetScan was used to predict the possible downstream effectors of *miR-140-3p* (Additional file 4: Fig. S4). Of the predicted target genes, *plxnb1* was selected as a candidate likely to influence bone formation. Plexin B1 has been reported to modulate osteogenesis by inhibiting the osteogenesis of osteoblasts and is activated by semaphorin 4D (sema4D), which is secreted by osteoclasts. To examine the relationship between *plxnb1* and *miR-140-3p*, *miR-140-3p* mimics and inhibitor were transfected into N-BMSCs and DM-BMSCs, respectively (Fig. 6A). The results indicated that DM-BMSCs expressed a greater quantity of plexin B1 in contrast to N-BMSCs. However, when transfected with *miR-140-3p* mimics, the levels of plexin B1 expressed in DM-BMSCs and N-BMSCs decreased considerably. Consistent with Western blotting analysis, qPCR demonstrated that *miR-140-3p* mimics substantially reduced *plxnb1* expression (Fig. 6B). To further ascertain the relationship and interaction between *plxnb1* and *miR-140-3p*, a dual Rluc/Fluc luciferase reporter plasmid containing the wildtype 3′-UTR of *plxnb1* (pSI-Check2-Plxnb1-3UTR) was generated (Fig. 6C). HEK293T cells were transfected with pSI-Check2-*Plxnb1*-3UTR WT/MU dual Rluc/Fluc luciferase reporter plasmids and a *miR-140-3p* mimics/negative control. The results demonstrated that *miR-140-3p* significantly inhibited the luciferase signal caused by *plxnb1* expression (Fig. 6D). However, the inhibition was largely abolished when four crucial nucleotides in the putative binding site for *miR-140-3p* were mutated (Fig. 6D). The results above suggest that *plxnb1* may be the downstream target of *miR-140-3p* for the regulation of osteogenesis.

Sema4D expressed by osteoclasts binds to its receptor Plexin-B1, resulting in the activation of the small GTPase RhoA, thereby inhibiting bone formation through inhibition of the insulin-like growth factor-1 (IGF-1) signaling pathway. Whether *miR-140-3p* regulates osteogenesis via *plxnb1* or not was then further explored. Firstly, N-BMSCs and DM-BMSCs were incubated with sema4D (Fig. 6E). The results demonstrate that the expression of p-RhoA and p-ROCK increased with increasing additions of sema4D, in a dose-dependent manner. In addition, p-IRS levels were reduced in these cells in a dose-dependent manner. Conversely, the expression of Runx-2, ALP, and Sp7 decreased due to increased sema4D in a dose-dependent manner in N/DM-BMSCs (Fig. 6H). The results indicate that activation of the sema4D/plexin B1/RhoA/ROCK signaling pathway may inhibit osteoblastogenesis in BMSCs. Secondly, the effects of *miR-140-3p* on the sema4D signaling pathway were investigated. *MiR-140-3p* mimics inhibited the effect of sema4D on the expression of p-RhoA and p-ROCK, and increased the expression of p-IRS in N/DM-BMSCs (Fig. 6F). In addition, *miR-140-3p* mimics reversed the effects of sema4D on Runx-2, ALP, and Sp7 expression and enhanced their osteogenic effects (Fig. 6I). These results suggest that *miR-140-3p* participates in osteoblast differentiation via modulation of the sema4D/plexin B1 signaling pathway. Lastly, BMSCs were incubated with anti-plexin B1 to elucidate the role of plexin B1 in osteogenesis. The results demonstrate that the expression of p-RhoA and p-ROCK decreased with increasing levels of anti-plexin B1 in a dose-dependent manner, despite treatment with sema4D (Fig. 6G). In addition, the expression of Runx-2, ALP, and Sp7 increased relative to the dose of anti-plexin B1 in N/DM-BMSCs (Fig. 6J). Thus, the results suggest that *plxnb1* is the direct downstream *mRNA* target of *miR-140-3p*, and that *miR-140-3p* can rescue osteogenesis by inhibition of the sema4D/plexin B1 signaling pathway in DM-BMSCs.

### Exosomes derived from BMSCs overexpressing miR-140-3p promote osteogenesis that heals bone defects when experiencing hyperglycemia

The therapeutic effects of *miR-140-3p* in regulating bone formation in DM rats were investigated by producing BMSC-specific exosomes overexpressing *miR-140-3p* and transplanting them into the bone defects of DM rats. To obtain exosomes overexpressing *miR-140-3p*, BMSCs were transfected with a lentivirus that overexpressed *miR-140-3p* (Additional file 5: Fig. S5). Exosomes overexpressing *miR-140-3p* were collected by ultracentrifugation. qPCR was used to confirm that, compared with N-Exos and DM-Exos, exosomes from BMSCs transfected with the lentivirus displayed high levels of *miR-140-3p* (Additional file 5: Fig. S5).

**Fig. 7:**
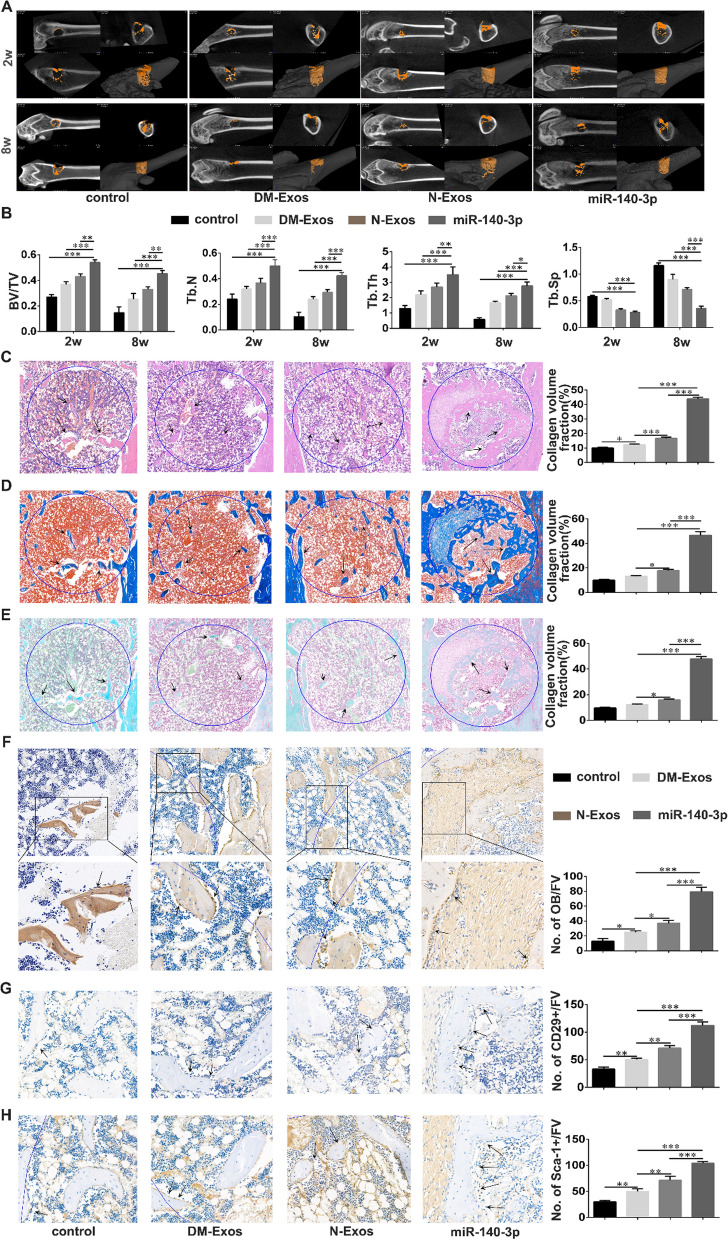
*MiR-140-3p* promotes bone formation in DM rats with bone defects. **A** Representative micro-CT images within a region of interest (ROI) comprising the bone defect after 2 and 8 weeks following surgery in the bone defect of normal rats and those with diabetes mellitus after treatment with N-Exos, DM-Exos, and Exos overexpressing *miR-140-3p*. n = 5 in each group. **B** Quantitative analysis of new bone formation from micro-CT imaging in DM rats with bone defects after 2 and 8 weeks after transplantation with N-Exos, DM-Exos, or Exos overexpressing *miR-140-3p*. BV: bone volume; TV: tissue volume; Tb,N: trabecular number; Tb.Th: trabecular thickness; Tb.Sp: trabecular Spacing. n = 5 in each group. **C**–**E** Histological analysis of decalcified sections of bone defects in diabetic rats treated with N-Exos, DM-Exos, or Exos overexpressing *miR-140-3p*. HE/Masson’s/Safranin O/fast green staining of the new bone formation within the defect. Black arrows indicate new bone stained in light pink/blue/green within the 3 mm-diameter defect marked as a circle. Quantitative analysis of new bone formation was performed using ImageJ software. n = 5 in each group. **F**–**H** Immunohistological analysis of osteoblasts and BMSCs in diabetic rats treated with N-Exos, DM-Exos, or Exos overexpressing *miR-140-3p*. Collagen-1 and CD29/Sca-1 staining of osteoblasts and BMSCs in sections containing the bone defect. Black arrows indicate osteoblasts and BMSCs on the bone surface. n = 5 in each group; *p < 0.05; **p < 0.01; ***p < 0.001. Data represent means ± SD

DM rats with bone defects were transplanted with 240 μg N-Exos, DM-Exos, or exosomes overexpressing *miR-140-3p* (140-Exos). Micro-CT was used to quantify bone mass in the different groups (Fig. 7A, B). The results demonstrate that although each exosomal treatment increased bone mass, the rats in which 140-Exos transplanted experienced an enhancement in bone mass than that observed in the N/DM-Exos transplanted rats after both 2 and 8 weeks, as manifested in the BV/TV values. Trabecular number and trabecular thickness increased after all exosome treatments compared with the control group, although their values were greater in the 140-Exos treatment compared with those for the N and DM-Exos treatments. Trabecular spacing declined in the exosome treatment groups compared with values in the control group. The data described above indicate that 140-Exos promoted bone defect remodeling which demonstrated the osteogenic effect of *miR-140-3p*.

In hematoxylin and eosin-stained sections, the extent of light pink collagen in the bone defect rats with Exos treated was greater than in control rats. Consistent with the findings above, the volume of collagen in the 140-Exos rats was highest in the N-Exos and DM-Exos transplantation groups (Fig. 7C). In the Masson’s-stained sections, the volume of mineralized bone tissue in the 140-Exos transplantation group was significantly greater than in the N/DM-Exos groups. In addition, the volume of mineralized bone tissue in the control rats was less than that observed in the exosome-treated rats (Fig. 7D). Safranin O/Fast green staining demonstrated that the volume of mineralized bone in 140-Exos was larger than that observed in the N/DM-Exos groups (Fig. 7E). Immunohistochemical staining of collagen-1 indicated that the numbers of osteoblasts in the control group were the least of all four groups while the numbers of osteoblasts in the 140-Exos group were greatest of the N/DM/140-Exos groups (Fig. 7F). The expression of the BMSC markers CD29/Sca-1 indicated that there were fewer BMSCs on the bone surface in the N/DM-Exos treatment groups than in the 140-Exos treated group, but more than in the control group (Fig. 7G, H). The data above indicate that 140-Exos significantly accelerated bone formation by increasing the numbers of BMSCs and osteoblasts in DM rats, resulting in greater mineralized bone formation and bone defect healing.

## Discussion

Delayed diabetic bone healing already represents an intractable medical problem, as current strategies are functionally limited. In the present study, a series of in vitro and in vivo studies were performed to determine whether BMSC-derived exosomal *miR-140-3p* promotes bone formation, and thus confirm the paradigm in which *miRNA* regulates the homeostatic mechanism of the local bone environment.

We found that DM-Exos and N-Exos derived from BMSCs containing *miRNA* modulated bone formation both in vivo and in vitro. In in vitro studies, exosomes from N-BMSCs and DM-BMSCs were found to promote osteoblastogenesis and mineralization in the BMSCs from both normal and DM rats, although N-Exos had a greater osteogenesis effect on osteoblastogenesis than did DM-Exos. Similarly, both N-Exos and DM-Exos enhanced bone regeneration in a femoral defect model in normal and DM rats, although the bone regenerative effect of N-Exos was greater than DM-Exos in vivo.

Bone modeling and remodeling describe processes in which bones are shaped or reshaped by the independent action of osteoblasts and osteoclasts. BMSCs not only function as progenitors of osteoblasts but also support osteoclast differentiation [28]. Diabetes has been shown to alter the properties of bone and impair bone repair in both humans and animals. There is accumulating evidence that diabetes mellitus decreases bone formation and the osteogenic capability of BMSCs [4]. DM is a chronic metabolic disease leading to high blood glucose levels which contribute to the production of advanced glycation end products (AGEs), reactive oxygen species, and tumor necrosis factor [3, 29, 30]. AGEs bind to specific receptors on BMSCs and inhibit BMSC differentiation, proliferation, and migration [31]. Additionally, AGEs increase BMSC apoptosis via MAPK signaling and oxidative stress [32, 33]. With high levels of inflammatory factors and the inhibitory action of hyperglycemia, the capacity of BMSCs to form bone has been shown to become downregulated with increased apoptosis, leading to a disruption of bone coupling and remodeling [34, 35].

Exosomes are derived from a variety of cell types and transfer into target cells whereby various functions are activated. Increasing numbers of reports indicate that stem cell-derived exosomes have the capability to promote bone formation [36]. As exosomes from different cell types provide a variety of therapeutic effects due to their specific cargos, we believe that exosomal therapy has considerable potential for clinical translation [37, 38]. In the present study, exosomes were extracted from BMSC-conditioned media by ultracentrifugation and identified from other extracellular vesicles by their characteristic diameter of 30–100 nm, as measured by NanoSight nanoparticle analysis and morphology by electron microscopy. Additionally, the exosome-specific marker TSG101 and extracellular vesicle-related protein markers HSP70, CD63, and CD9 were measured by Western blotting analysis. Together, the results indicate that the extracellular vesicles were indeed exosomes [39–41]. Moreover, we used transwell culture and transfection to demonstrate that exosomal *miRNAs* could directly transfer into recipient cells. We firstly transfected BMSCs with Cy3-labeled *miR-223* and transplanted them into the upper chamber of a transwell insert with untransfected BMSCs placed in the lower chamber. The red fluorescence of Cy3-labeled *miR-223* in the BMSCs in the lower chamber was detected. Untransfected BMSCs were then incubated with exosomes labeled with PKH26 fluorescent dye. This demonstrated that non-transfected BMSCs absorbed fluorescent-labeled exosomes, displaying green fluorescence. *miR-223* expression also increased in non-transfected BMSCs, which was inhibited by prior treatment of BMSCs with GW4869 (an inhibitor of EV secretion). The results demonstrated that exosomes and exosomal *miRNA* could be directly transferred into recipient cells.

The bone environment has also been found to be altered in individuals suffering from T2DM, which has a negative impact on bone formation. We found that DM-BMSC derived-Exos promoted bone formation and increased bone mass in normal rats compared with control rats, although the osteogenic capability of DM-Exos was significantly impaired compared with that of N-Exos treated rats. Nevertheless, N-Exos promoted bone regeneration and increased bone volume/tissue volume in DM rats compared with DM-Exos transplanted rats. Similarly, the volume of collagen in DM-Exos-treated normal rats was less than that observed in the N-Exos treatment group, while DM-Exos decreased the number of osteoblasts and BMSCs in normal rats compared with the N-Exos group. As described above, N-Exos enhanced the volume of collagen in DM rats and the numbers of osteoblasts and BMSCs compared with the DM-Exos group. In addition, exosomes promoted mineralization generated in BMSCs, although the osteogenic capability of N-Exos was superior to that of DM-Exos. The results indicate that DM-BMSC-derived exosomes impaired bone regeneration. The results were consistent with previous studies [15]. However, few studies have concentrated on differences between DM-BMSC-derived exosomes and those from normal BMSCs. Thus, we explored the differences here.

Exosomal miRNAs have become a focus of recent research attention due to their numerous roles in regulation [40, 42]. *MiRNAs* and RNA-induced silencing complexes (RISCs) are packaged into exosomes and transferred to recipient cells to silence *mRNAs* [43]. Numerous research studies have revealed that a number of bone-derived exosomal *miRNAs* participate in bone remodeling. There are 43 exosomal *miRNAs* highly expressed in MC3T3-E1 cells during the osteogenesis process, such as *miR-30d-5p* and *miR-133b-3p* [44, 45]. Moreover, *miR-135b*, *miR-203*, and *miR-299-5p* are upregulated in human-BMSCs derived exosomes during osteogenesis [46–48]. Thus, in the present study, we aimed to identify the differences in exosomal *miRNAs* between N-Exos and DM-Exos. To investigate the effect of DM on changes to *miRNAs*, high-throughput sequencing was used to determine the identity of differentially expressed exosomal *miRNAs*. More than 600 *miRNAs* were sequenced and quantified, from which *miR-140-3p*, *miR-34c-5p*, *miR-99a-5p*, and *miR-27a-3p* were found to be in significantly different abundance in DM-Exos compared with N-Exos. These have been identified in previous studies for their ability to promote osteogenesis [49–51]. BMSCs were incubated with *miRNA* mimics and inhibitor to ascertain the osteogenic effects of these *miRNAs*. The results indicated that *miR-140-3p* displayed the greatest promotion of BMSC differentiation compared with other *miRNAs*. In addition, the expression of *miR-140-3p* was lower in DM-BMSC, DM-ADSC, and DM-BMSC Exos, suggesting that the inhibition by DM of osteogenesis in BMSCs may decrease levels of *miRNA* in BMSCs. To further demonstrate the impact of *miR140-3p* on osteogenesis, we transfected BMSCs with *miR-140-3p*, the results of which demonstrated that *miR-140-3p* mimics significantly promoted *Col-1, Runx-2, Sp7*, and *ALP* expression in DM-BMSCs and N-BMSCs compared with the control and inhibitor groups. Western bloting analysis and qPCR indicate that *miR-140-3p* played an important role in the differentiation of BMSCs. Similarly, Rakefet Pando found that chondrocyte-specific *miR-140-3p* displayed the greatest expression in mature EGP(epiphyseal growth plate), and was one of only a few *miRNAs* that were significantly reduced in expression following restricted nutrition. Additionally, SIRT1 levels increased significantly in nutrition-restricted states. Their study indicated that the expression of *miR-140-3p* was positive during nutrition-induced catch-up growth in the epiphyseal growth plate (EGP) [52]. Moreover, consistent with the findings of the present study, S. Takahara found that *miR-140-3p* levels were significantly lower in DM rats after 14 days compared with control rats [53]. Furthermore, *miR-140-3p* was found to be significantly highly expressed in rats undergoing bone fracture healing compared with those that were not healing [54]. Together, the results established that *miR-140-3p* exerted an osteogenic effect on BMSCs.

To further determine the mechanisms by which *miR-140-3p* was involved in the regulation of bone regeneration, we explored the target *mRNAs* of *miR-140-3p*. Theoretically, *miRNAs* can have more than one *mRNA* target, and there could be more than one *miRNA* directed against an mRNA molecule [55]. We used TargetScan software to analyze the sequenced *mRNA* molecules in BMSCs. This analysis revealed that several *mRNA* molecules represented downstream effectors of *miR-140-3p*. Of the numerous candidates, *plxnb1* emerged as having a positive association with bone formation and osteogenesis. Plexin B1 has been reported to be a receptor of sema4D, which is secreted by osteoclasts. Sema4D binds to plexin B1 and inhibits osteogenesis in osteoblasts, reducing bone formation [56–58]. To confirm that *plxnb1* is the effector of *miR-140-3p*, dual Rluc/Fluc luciferase reporter plasmids containing the wildtype 3′-UTR of PLXNB1 (*pSI-Check2-Plxnb1-3UTR*) were generated, the results showing that *miR-140-3p* significantly inhibited luciferase activity due to *plxnb1*. However, this inhibition was largely abolished when four crucial nucleotides were mutated in the putative binding site of *miR-140-3p*. In addition, the expression of plexin B1 was significantly lower in BMSCs transfected with *miR-140-3p*. The results demonstrate that *miR-140-3p* promoted osteogenesis by inhibition of the plexinB1/RhoA/ROCK signaling pathway.

In summary, the findings indicate that considerable differences exist in the *miRNAs* contained in N-Exos compared with DM-Exos, which supports the hypothesis that BMSC-derived exosomal *miR-140-3p* functions as an important regulator of osteoblastogenesis. Moreover, administration of *miR-140-3p* upregulated exosomes to the bone marrow microenvironment may provide a potential therapeutic strategy for diabetes-induced bone fracture/defect.

## Conclusion

Taken together, our results suggest that diabetes mellitus impairs BMSCs differentiation and bone regeneration by decreasing the levels of miR-140-3p in BMSCs and exosomes, which lead to the increased expression of plexin B1 in vivo and vitro. These findings provide new insights into the molecular mechanism of impaired bone regeneration and BMSCs differentiation in T2DM patients and strategies to remediation.

## Materials and methods

### Animals and treatment

One hundred 4-week-old male Sprague–Dawley rats were obtained from the Animal Center of the Air Force Military Medical University (Xi’an, China). All animal experiments and care protocols were reviewed and approved by the Animal Research Committee of the Air Force Military Medical University (SCXK-2019–001). All animals were provided ad libitum access to food and water prior to the experiments. Rats were fed a high fat and high carbohydrate diet (HFD) for one month after which streptozotocin (Sigma-Aldrich, St. Louis, MO, USA, 40 mg/kg) dissolved in 10 mmol/L citrate buffer (pH 4.5) was injected intraperitonealy once into the rats, which were then fasted for 2 h, then allowed to eat freely. After 7 days, blood glucose concentrations were tested via a blood glucometer. Rats with blood glucose levels higher than 16.7 mmol/L were considered to be diabetes mellitus positive.

### Bone defect model

A bone defect model was established in accordance with a previous study. Nine-week-old male Sprague–Dawley rats were anesthetized using sodium pentobarbitone (3% m/m). A 1.0-cm-long incision was created along the femur in the sagittal plane on right side. A bone defect was created in the metaphysis by drilling a 3 mm-diameter hole. Exosomes (240 μg) were thoroughly mixed with Matrigel (Corning, NY, USA, 356,230) and placed on ice prior to injecting into the bone defects. Forty rats were randomly allocated into the following four groups: (a) matrigel group (control group; n = 10); (b) matrigel mixed with N-Exos (N-Exos group; n = 10); (c) matrigel mixed with DM-Exos (DM-Exos group; n = 10); and (d) matrigel mixed with Exos overexpressing *miR-140-3p* (140-Exos group; n = 10).

### Micro-CT analysis

Rats were anesthetized with 3% sodium pentobarbitone allowing the microarchitecture of the femoral cavities to be examined by micro-CT. Bone images were reconstructed using an isotropic voxel size of 10 μm. Bone volume/total volume, trabecular thickness, trabecular number, and trabecular spacing values were recorded to evaluate bone formation. Three-dimensional imaging and the reconstruction of the microarchitecture were performed using an Inveon Research Workplace 2.2 (SIEMENS Healthineers, Berlin, Germany).

### BMSC isolation and treatment

BMSCs were harvested from both femurs of the rats and sorted using a FACS Aria II flow cytometer (BD Biosciences, New Jersey, USA). Bone marrow cells were flushed from the bone marrow cavity after which a red blood cell lysis buffer was used to eliminate red blood cells. The remaining cells were then enumerated. Positivity toward a PE-conjugated mouse monoclonal antibody against integrin beta 1 (Abcam, Cambridge, UK, ab218273) and a FITC-conjugated rat monoclonal antibody against Sca1/Ly6A/E (Abcam, ab268016) was utilized to separate BMSCs from other bone marrow cells. The sorted cells were transfected with miRNA mimics/inhibitor, or treated with siRNA or an agonist for additional experiments. In addition, exosomes (2 μg) were added to the culture medium of BMSCs (1 × 10^5^).

### Exosomes isolation, identification

Exosomes were isolated using ultracentrifugation, as described previously [39]. Cell culture supernatants were centrifugated at 300 g for 10 min, 2000 g for 30 min, 10,000 g for 5 min at 4 ℃, then finally at 100,000 g for 4 h at 4 ℃ using an SW28 rotor (Beckman Coulter, Fullerton, CA, USA). The pelleted exosomes were collected then subsequently resuspended in PBS. Western blotting was used to quantify specific marker proteins in the exosomes, including HSP70, TSG101, CD63, and CD9. The size distribution of the exosomes was measured by nanoparticle tracking analysis (ZetaView PMX 110,Meerbusch, Germany) and their morphology was assessed using transmission electron microscopy (FEI Tecnai Spirit, Oregon, USA).

### Immunohistochemistry

Harvested rat femurs were fixed in 4% paraformaldehyde (PFA) then decalcified in ethylenediaminetetraacetic acid (EDTA; 10%, pH 7.0) prior to embedding in paraffin. Samples were sliced into 5 μm-thick sections using a microtome in a plane parallel to that of the bone defect cavity. The sections were then incubated in dimethylbenzene I and II, twice in 100% ethanol, twice in 95% ethanol, then in 90% ethanol and 80% ethanol, respectively. Endogenous peroxidase activity was eliminated with 3% H_2_O_2_ after which samples were incubated with goat serum at room temperature for 30 min. Finally, the samples were incubated with a specific primary antibody for 12 h at 4 °C, from the following panel: mouse monoclonal anti-collagen I (Col-1;ab270993, Abcam); rabbit polyclonal anti-CD29 (GeneTex,California,USA,GTX128839); rabbit anti-mouse Sca-1 (Biolegend, California,USA,108,103) and Rabbit IgG(ab125938) which was regarded as negative control in IHC. Sections were then incubated with a biotinylated secondary antibody or conjugated with horseradish peroxidase (HRP) at room temperature for 30 min, after which color was developed with diaminobenzidine (DAB) and the sections counterstained with hematoxylin. Images were acquired following observation by light microscopy (Olympus BX53, Tokyo, Japan).

### Histological analysis

The harvested rat femurs were fixed in PFA (4%) then decalcified in EDTA (10%, pH 7.0), respectively, prior to embedding in paraffin. The tissue was sliced into 5 μm-thick sections using a microtome in a plane parallel to that of the bone. The sections were deparaffinized then rehydrated, as reported previously[59]. The sections were then stained with hematoxylin and eosin, Masson’s trichrome, and Safranin O/fast green to evaluate new bone formation.

### Alizarin staining

Alizarin red S staining was used to evaluate mineralization. The BMSCs were fixed in 4% paraformaldehyde for 30 min, then stained with alizarin red S for 20 min. Images were obtained via an Olympus BX53 microscope (Olympus, Tokyo, Japan). The extent of calcium deposition was assessed in each group to determine osteogenesis.

### Western blotting

Cells or exosomes were lysed in RIPA buffer and the concentration of protein in the lysates was measured using a bicinchoninic acid (BCA) kit. The lysates were diluted with loading buffer (5 ×) then heated to 100℃ for 10 min. Individual proteins were separated using 10% sodium dodecyl sulfate polyacrylamide gel electrophoresis (SDS-PAGE) after which they were transferred onto polyvinylidene fluoride (PVDF) membranes PVDF, Millipore, MA, USA). Non-specific binding was blocked by incubating the membranes with 5% nonfat milk for 2 h at room temperature and the identity of proteins evaluated by incubation at 4 ℃ overnight with the following primary antibodies (Abcam): HSP70 (1:1000, ab2787), TSG101 (1:5000, ab125011), CD63 (1 μg, ab193349), CD9 (1:2000, ab92726), col-1 (1:1000, ab270993), Runx-2 (1:1000, ab76956), Sp7 (1:1000, ab209484), and ALP (1:1000, ab229126). Membranes were then incubated with either an anti-rabbit IgG or anti-mouse IgG (1:10,000, Boster Bio-Technology, Wuhan, China) HRP-conjugated secondary antibody, as appropriate, for 2 h at room temperature. The density of the immunoreactive bands was analyzed using a ChemiDoc XRS (Bio-Rad, Hercules, CA, USA) and quantified using Quantity One version 4.1.0 software (Bio-Rad).

### qPCR

Total RNA from exosomes or BMSCs was extracted using Trizol reagent ((Invitrogen, California, USA)). A One Step SYBR® PrimeScript™ qPCR kit (TaKaRa Bio, Otsu, Japan) was used to synthesize cDNA, in accordance with the manufacturer’s instructions. Quantitative real-time PCR (qPCR) was performed using SYBR® Premix Ex Taq™ (TaKaRa) in a Bio-Rad CFX96™ real-time PCR system using the following thermocycling conditions: pre-denaturation at 95 °C for 5 s, followed by 40 cycles of denaturation at 95 °C, 10 s; annealing at 57 °C, 20 s; and extension at 72 °C, 20 s. The relative expression of the specified genes was calculated using the 2^−ΔΔCT^ method after normalization to GAPDH expression.

In addition, miRNA was quantified by synthesizing cDNA using a Sangon Biotech miRNA First Strand cDNA synthesis (tailing reaction) kit (B532451), in accordance with the manufacturer’s protocol then performing qPCR using a Sangon Biotech microRNA qPCR kit with SYBR Green (B532461) in a Bio-Rad CFX96™ real-time PCR detection system (Bio-Rad) using the following thermocycling conditions: pre-denaturation at 95 °C for 30 s, followed by 40 cycles of denaturation at 95 °C, 5 s; annealing at 60 °C, 30 s; and extension at 72 °C, 30 s. The relative expression of each gene was calculated using the 2^−ΔΔCT^ method after normalization to U6 expression (Table 1).

**Table 1:** The sequences of primers

Primer	Sequence
Runx-2 F	CCCAGCCACCTTTACCTACA
Runx-2 R	TGGGAACTGATAGGATGCTG
ALP F	CACGGCGTCCATGAGCAGAAC
ALP R	CAGGCACAGTGGTCAAGGTTGG
Osterix F	CTGCAACTGGCTTTTCTGC
Osterix R	CAGCTCCTTAGGGCCACTT
Plxnb1 F	CGAGGCGGAGGAGTGGATGG
Plxnb1 R	CGAGGCTGGACAGGAGGATGAG
GAPDH F	GCTGAGTATGTCGTGGAGT
GAPDH R	GTTCACACCCATCACAAAC
miR-140-3p	TATACCACAGGGTAGAACCACGG
U6 F	CTCGCTTCGGCAGCACA
U6 R	AACGCTTCACGAATTTGCGT

### Firefly luciferase and Renilla luciferase activity assay

HEK293 cells were seeded in 96 well plates an cultured to 50%-70% confluence prior to transfection. A 0.16 μg quantity of plasmids consisting of the 3′-untranslated region (UTR) of plxnb1 with a firefly luciferase reporter and psiCHECK-2 with Renilla luciferase reporter were mixed with 10 μL DMEM and 5 pmol rno-miR-140-3p/Negative Control (N.C.) at room temperature for 5 min, termed solution A, then 10 μL DMEM were mixed with 0.3 μL Lipofectamine MessengerMax (ThermoFisher, Massachusetts, USA, LMRNA001), termed solution B. Solution C was formed by blending A with B and incubating at room temperature for 20 min. The HEK293 cells were then incubated with solution C for 6 h after which luciferase luminescent intensity was measured using a Promega dual-luciferase assay kit, in accordance with the manufacturer’s protocol.

### Statistical analysis

All data are presented as means ± standard deviation (SD). Three independent experiments were analyzed using SPSS version 15.0 software. Statistical significance was determined by one-way ANOVA, while multiple comparisons were performed using a Student–Newman–Keuls t-test. P < 0.05 was considered statistically significant.

## Supplementary Information

**Additional file 1: Figure S1.** Characteristics of Exosomal *miRNAs*.

**Additional file 2: Figure S2.** Impairment of BMSC differentiation in vitro by DM-Exos.

**Additional file 3: Figure S3.** Different miRNAs Expression between DM-Exos and N-Exos.

**Additional file 4: Figure S4.** TargetScan predicted the possible downstream effectors of *miR-140-3p*.

**Additional file 5: Figure S5.** The expression of miR-140-3p in BMSCs and Exos.

## Data Availability

The datasets generated and/or analysed during the current study are available in the [baidu] repository, [https://pan.baidu.com/s/1Pn0Kp_WXk_PJrcjU962o4g].
