# Supplementary material for: Increased BMSC exosomal miR-140-3p alleviates bone degradation and promotes bone restoration by targeting Plxnb1 in diabetic rats
Source: J Nanobiotechnology. 2022 Mar 2;20:97. doi: 10.1186/s12951-022-01267-2 (PMC8889728; doi:10.1186/s12951-022-01267-2)
Supplement: Supplementary file 1 — Additional file 1: Figure S1. Characteristics of Exosomal miRNAs. [file 12951_2022_1267_MOESM1_ESM.docx]

Additional file 1


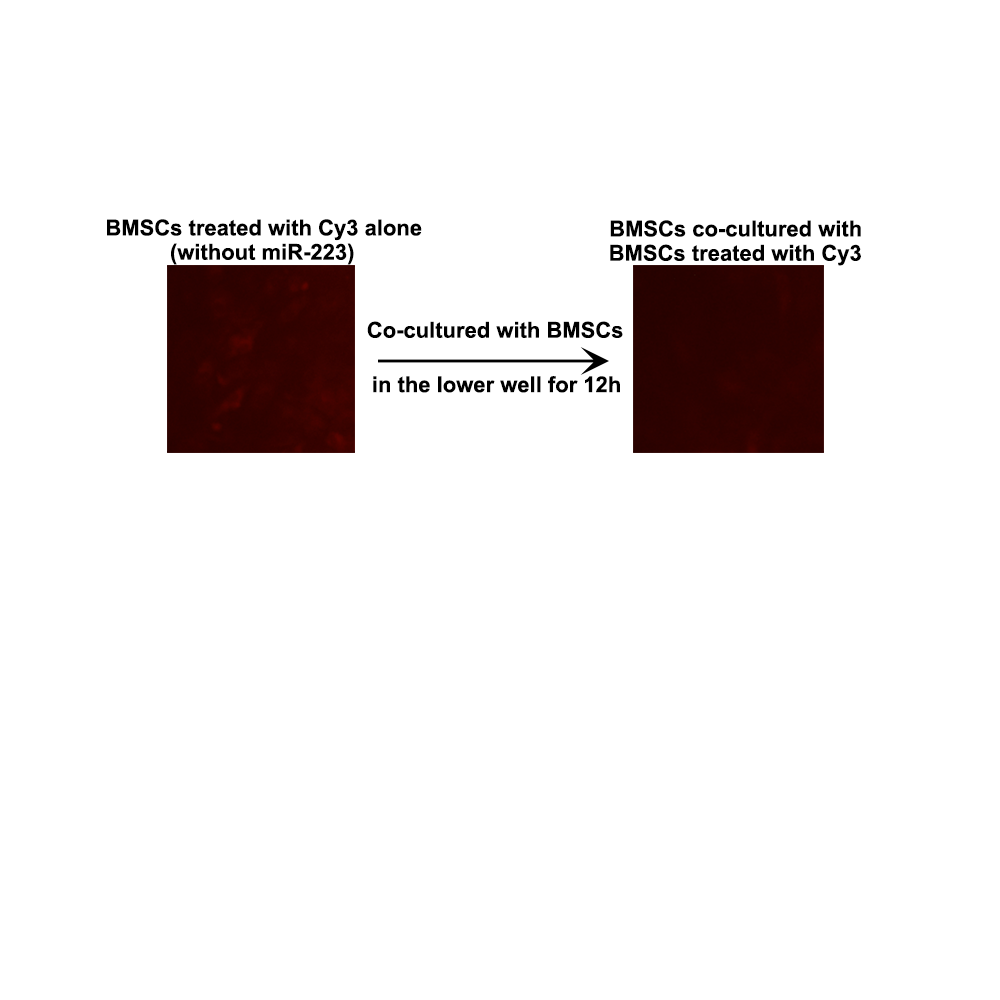


**Figure S1. Characteristics of Exosomal *miRNAs***

The appearance of Cy3 red fluorescence in BMSCs seeded in lower well was examined after co-culture with BMSCs in upper chamber treated with Cy3 dye (without *miR-223* mimic) for 12h. n=3.
