# Supplementary material for: Increased BMSC exosomal miR-140-3p alleviates bone degradation and promotes bone restoration by targeting Plxnb1 in diabetic rats
Source: J Nanobiotechnology. 2022 Mar 2;20:97. doi: 10.1186/s12951-022-01267-2 (PMC8889728; doi:10.1186/s12951-022-01267-2)
Supplement: Supplementary file 2 — Additional file 2: Figure S2. Impairment of BMSC differentiation in vitro by DM-Exos. [file 12951_2022_1267_MOESM2_ESM.docx]

Additional file 2


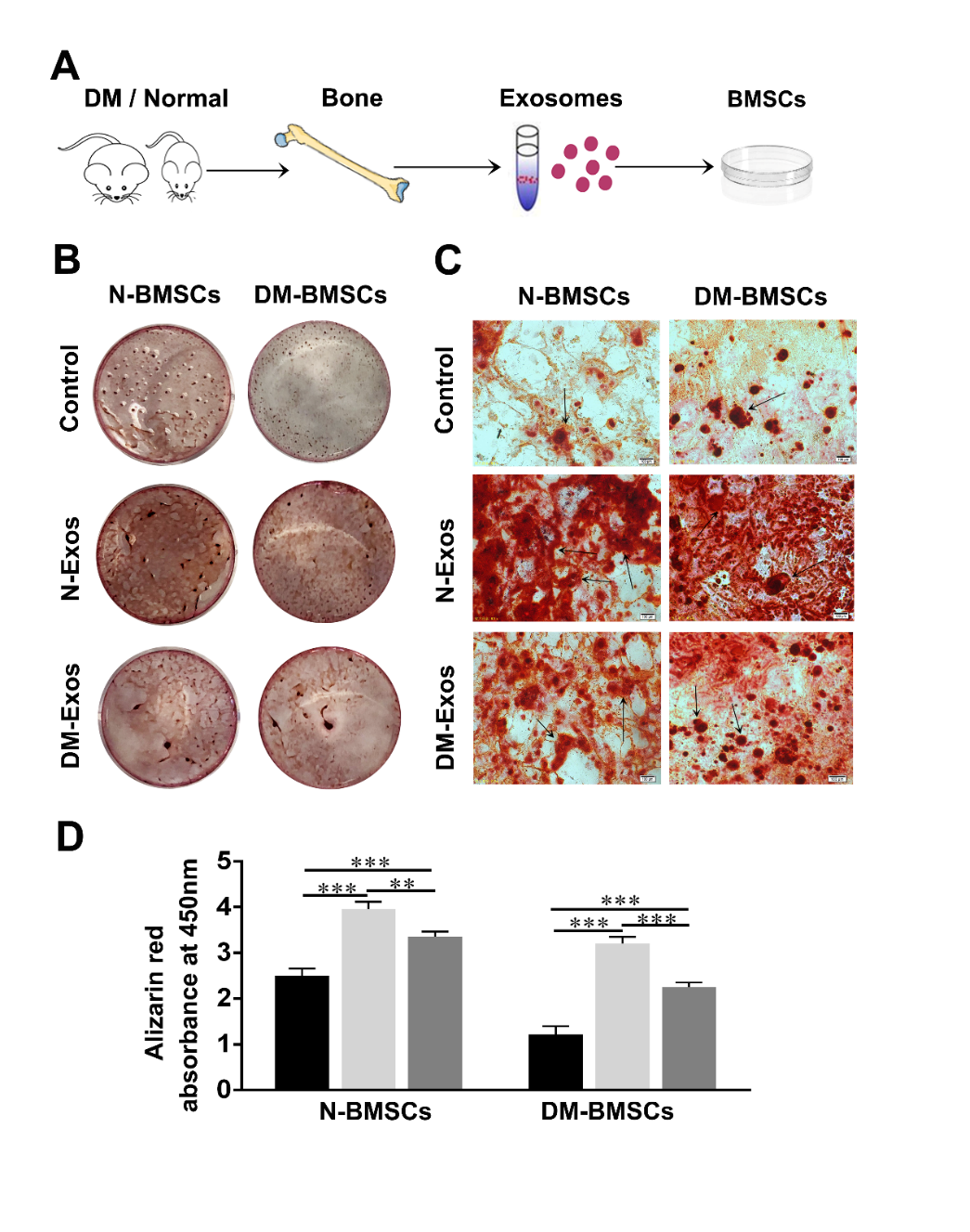


**Figure S2. Impairment of BMSC differentiation *in vitro* by DM-Exos**

(A) N-Exos and DM-Exos were cultured with normal rat BMSCs or DM rat BMSCs for 14 days *in vitro*.

(B,C) Osteogenic differentiation of BMSCs examined by Alizarin red staining on day 14. n=3 in each group.

(D) Calcium mineralization quantified by optical density using a microplate reader. n=3 in each group. **p < 0.01; ***p < 0.001. Data represent means ± SD.
