# Supplementary material for: Increased BMSC exosomal miR-140-3p alleviates bone degradation and promotes bone restoration by targeting Plxnb1 in diabetic rats
Source: J Nanobiotechnology. 2022 Mar 2;20:97. doi: 10.1186/s12951-022-01267-2 (PMC8889728; doi:10.1186/s12951-022-01267-2)
Supplement: Supplementary file 3 — Additional file 3: Figure S3. Different miRNAs Expression between DM-Exos and N-Exos. [file 12951_2022_1267_MOESM3_ESM.docx]

Additional file 3


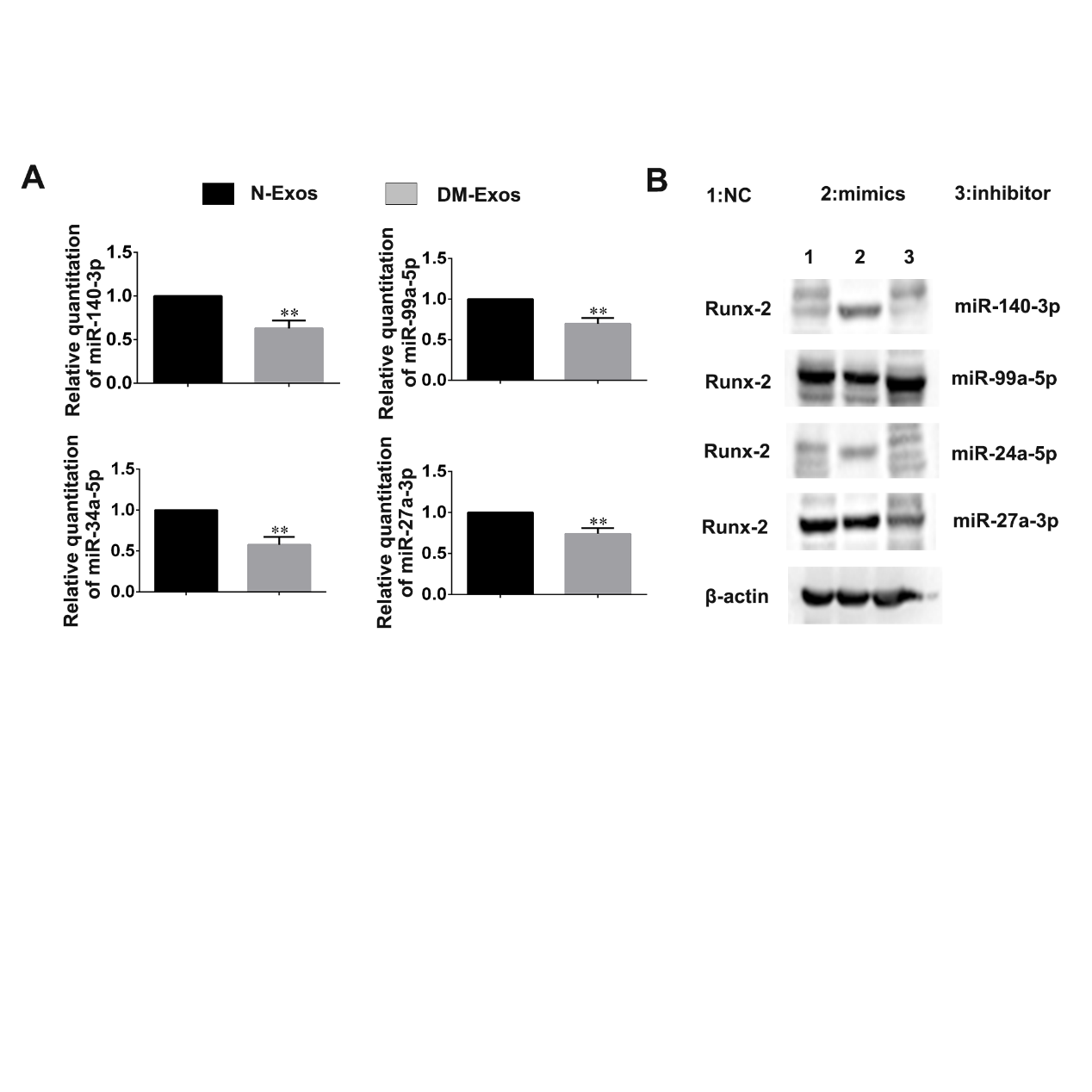


**Figure S3. Different miRNAs Expression between DM-Exos and N-Exos**

(A) *MiR-140-3p/99a-5p/34a-5p/27a-3p* differential expression level between N-BMSCs and DM-Exos. (B) Western blotting determined the Runx-2 expression in BMSCs treated with *miR-140-3p/99a-5p/34a-5p/27a-3p* NC/mimics/inhibitor. N=3 in each group. **p < 0.01. Data are presented as the mean ± SEM.
