# Supplementary material for: Increased BMSC exosomal miR-140-3p alleviates bone degradation and promotes bone restoration by targeting Plxnb1 in diabetic rats
Source: J Nanobiotechnology. 2022 Mar 2;20:97. doi: 10.1186/s12951-022-01267-2 (PMC8889728; doi:10.1186/s12951-022-01267-2)
Supplement: Supplementary file 4 — Additional file 4: Figure S4. TargetScan predicted the possible downstream effectors of miR-140-3p. [file 12951_2022_1267_MOESM4_ESM.docx]

Additional file 4





**Figure S4. TargetScan predicted the possible downstream effectors of *miR-140-3p***
