# Supplementary material for: Increased BMSC exosomal miR-140-3p alleviates bone degradation and promotes bone restoration by targeting Plxnb1 in diabetic rats
Source: J Nanobiotechnology. 2022 Mar 2;20:97. doi: 10.1186/s12951-022-01267-2 (PMC8889728; doi:10.1186/s12951-022-01267-2)
Supplement: Supplementary file 5 — Additional file 5: Figure S5. The expression of miR-140-3p in BMSCs and Exos. [file 12951_2022_1267_MOESM5_ESM.docx]

Additional file 5


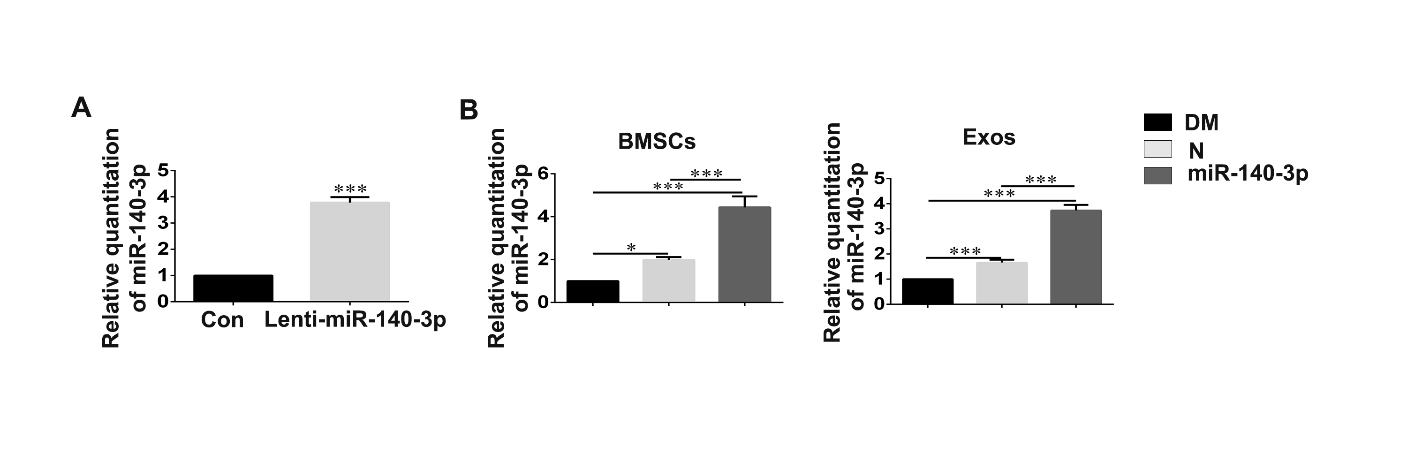


**Figure S5. The expression of miR-140-3p in BMSCs and Exos**

(A) Expression of miR-140-3p in BMSCs transfected with lenti-miR-140-3p. (B) The levels of miR-140-3p among DM-BMSCs, N-BMSCs and BMSCs transfected with lenti-miR-140-3p and the paralleled Exos. N=3 in each group. *p < 0.05; ***p < 0.001. Data are presented as the mean ± SEM.
